# Circulating DPP3 limits efficacy of angiotensin II infusion in septic shock

**DOI:** 10.1186/s13054-026-05971-5

**Published:** 2026-03-25

**Authors:** Dirk van Lier, Karine Santos, Oliver Hartmann, Matthijs Kox, Peter Pickkers

**Affiliations:** 1 Department of Intensive Care Medicine and Radboud Center for Infectious Diseases (RCI), Radboud university medical center, Nijmegen, The Netherlands; 2 4TEEN4 Pharmaceuticals GmbH, Hennigsdorf, Germany

## Dear editor

The hemodynamic response to infusion of angiotensin (Ang)-II in patients with catecholamine-resistant vasodilatory shock (CRVS) is variable. Various methods to select patients that show a more pronounced response have been described, including biomarkers (such as renin and the Ang-I/Ang-II ratio) or the initial increase in blood pressure [1, 2]. During shock, the cytosolic enzyme DPP3 is released into the bloodstream as a consequence of profound tissue perfusion perturbances, correlating with hemodynamic instability and impaired clinical outcomes [3]. As circulating (c)DPP3 can also degrade infused Ang-II, it may limit its therapeutic efficacy. Therefore, we investigated the relationship between cDPP3 concentrations and the efficacy of Ang-II infusion to increase blood pressure in a cohort of sepsis patients with CRVS.

Patients fulfilling sepsis-3 criteria with a noradrenaline infusion rate > 0.2 µg/kg/min were treated with 20–40 ng/kg/min Ang-II (AT-II therapy) within 6–48 h after initiation of noradrenaline therapy, dependent on its efficacy. Patients were deemed a responder to AT-II infusion if a > 25% reduction in norepinephrine infusion rate was achieved within 3 h.

cDPP3 concentrations were determined at baseline, and at 3 and 24 h after start of AT-II therapy using a luminescence immunoassay described previously [4]. Renin, Ang-I and Ang-II concentrations were determined using a mass spectrometry-based approach, in pre-chilled blood samples with immediate addition of a plasma protease inhibitor cocktail [5]. Ang-I/II ratios were calculated as a proxy of renin-angiotensin-aldosterone system (RAAS) dysfunction. Data are presented as median [IQR], Wilcoxon’s signed-rank tests were performed to assess differences between groups.

Ten patients were included, of whom six were responders to AT-II, with a reduction of norepinephrine infusion rate of 77% [49–90%] vs. 3% [-12-20%] % in non-responders. Responders had lower SOFA scores (9 [7–10) vs. 13 [11–15], *p* = 0.018), and a lower norepinephrine infusion rate at baseline (0.22 [0.20–0.30] vs. 0.50 [0.41–0.63) µg/kg/min, *p* = 0.019).

**Fig. 1:**
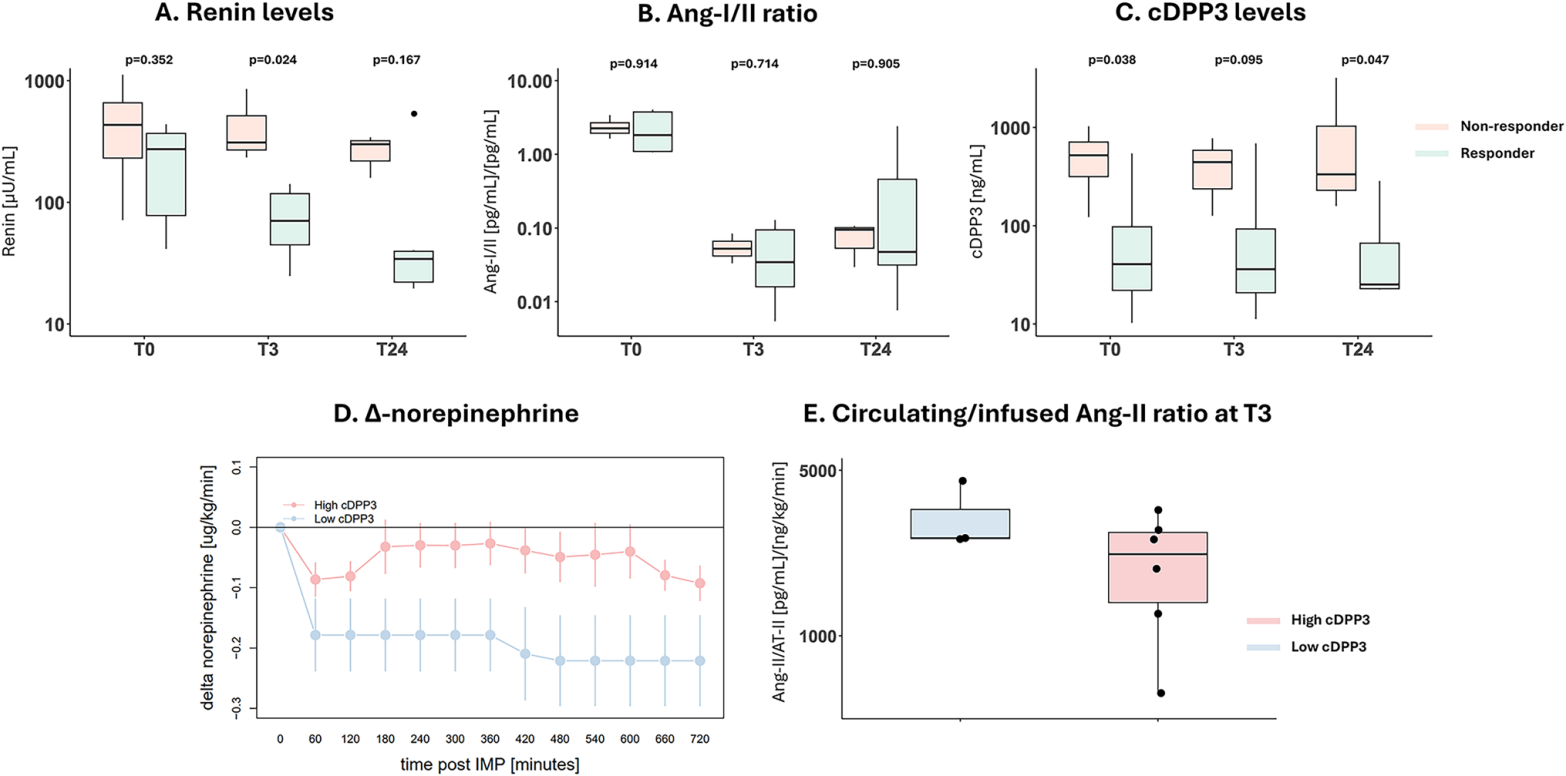
Panel **A**: Renin levels before (T0) and 3 and 24 h after start of angiotensin II infusion, in responders (reduction in norepinephrine infusion rate > 25%) and non-responders. Panel **B**: Angiotensin I/II ratios before and after start of angiotensin infusion in responders/non-responders. Panel **C**: cDPP3 levels before and after start of angiotensin infusion in responders/non-responders. Panel **D**: Norepinephrine dose (NED) change compared to baseline (T0) after start of angiotensin II infusion, in high/low cDPP3 subgroups based on a previously established cutoff of 40 ng/mL. Panel E: Angiotensin II infusion/circulating angiotensin II ratio in high/low cDPP3 subgroups 3 h after the start of angiotensin II infusion

Baseline renin concentrations were not significantly different between responders and non-responders;282 [95–368] vs. 446 [274–691] µU/mL, respectively, *p* = 0.352. Renin concentrations declined in both groups, although the decrease was significantly more pronounced in AT-II responders (Fig. 1A). No differences in baseline Ang-I/II ratios were found. A steep decline in Ang-I/II ratios was observed in both subgroups following initiation of AT-II therapy (Fig. 1B). Baseline cDPP3 concentrations were significantly lower in responders than in non-responders (44 [22–101] vs. 531 [355–728] ng/mL, respectively, *p* = 0.038), and a between-group difference persisted after initiation of AT-II therapy (Fig. 1C). Correspondingly, more pronounced reductions in norepinephrine infusion rate were observed in patients with low cDPP3 concentrations compared with those exhibiting high cDPP3 concentrations (Fig. 1D). Interestingly, the circulating Ang-II/infused AT-II ratio tended to be higher in the high cDPP3 subgroup, suggesting that although these patients received a higher infusion rate of AT-II, this did not result in a dose-proportional increase in circulating Ang-II concentrations (Fig. 1E), plausibly due to cDPP3-mediated breakdown of AT-II.

Our data suggest a causative pathophysiological role for cDPP3 in the development of CRVS. The attenuated efficacy of AT-II therapy to increase blood pressure in patients with high cDPP3 concentrations and the less-than dose proportional increases in circulating angiotensin II concentrations following initiation of AT-II therapy in these patients imply breakdown of Ang-II, confirming cDPP3’s mechanism of action. As cDPP3 degrades circulating Ang-II, it may attenuate the negative feedback mechanism whereby Ang-II suppresses renal renin production after RAAS function has been restored. This could account for the persistently elevated renin concentrations observed in non-responders.

Baseline renin concentrations in this pilot study were almost to two-fold higher than those reported in the ATHOS-III trial. This might explain why baseline renin concentrations were not associated with AT-II therapy responsiveness in the current study, as maximum renin responses were already present in both subgroups as a consequence of profound RAAS dysfunction. The absence of differences in Ang I/II ratios are plausibly explained by the large variability in angiotensin metabolite concentrations, which, combined with the small sample size, limited the chances of detecting statistically significant differences.

Interestingly, the authors of the ATHOS-III post-hoc analyses speculated on the possibility of intrinsic defects in ACE function, or unknown circulating inhibitory peptides as pathophysiological mechanisms explaining the vasopressor-resistant phenotype. Based on our results, cDPP3 appears to be a key inhibitory peptide in this process, reducing endogenous Ang-II responses to an extent that is relevant for the development of CRVS.

Our findings have several potentially relevant clinical implications. First, cDPP3 shows promise as a theragnostic biomarker in CRVS, with high concentrations predisposing for reduced efficacy of Ang-II (rescue) vasopressor therapy. Second, a cDPP3-blocking antibody named Procizumab, aimed at preventing cDPP3 deleterious effects, might be implemented as monotherapy to improve hemodynamic stability but also as an adjunctive therapy to improve efficacy of AT-II therapy. This drug is currently undergoing phase-1b/2a clinical testing [3] and results are eagerly awaited.

## Data Availability

Data is available upon reasonable request.

## Abbreviations

CRVS: Catecholamine resistant vasoplegic syndrome
DPP3: Dipeptidyl peptidase 3
cDPP3: Circulating dipeptidyl peptidase 3
Ang: Angiotensin
RAAS: Renin angiotensin aldosterone system
SOFA: Sequential organ failure assessment
